# Expression of ZIC family genes in meningiomas and other brain tumors

**DOI:** 10.1186/1471-2407-10-79

**Published:** 2010-03-03

**Authors:** Jun Aruga, Yayoi Nozaki, Minoru Hatayama, Yuri S Odaka, Naoki Yokota

**Affiliations:** 1 Laboratory for Behavioral and Developmental Disorders, RIKEN Brain Science Institute, Wako-shi, Saitama 351-0198, Japan; 2 Department of Neurosurgery, Hamamatsu University School of Medicine, 1-20-1 Handa-yama, Hamamatsu, Shizuoka 431-3192, Japan

## Abstract

**Background:**

Zic zinc finger proteins are present in the developing rodent meninges and are required for cell proliferation and differentiation of meningeal progenitors. Although human *ZIC *genes are known to be molecular markers for medulloblastomas, their expression in meningioma has not been addressed to date.

**Methods:**

We examined the mRNA and protein expression of human *ZIC1*, *ZIC2*, *ZIC3*, *ZIC4 *and *ZIC5 *genes in meningiomas in comparison to other brain tumors, using RT-PCR, analysis of published microarray data, and immunostaining.

**Results:**

*ZIC1*, *ZIC2 *and *ZIC5 *transcript levels in meningiomas were higher than those in whole brain or normal dura mater, whereas all five *ZIC *genes were abundantly expressed in medulloblastomas. The expression level of *ZIC1 *in public microarray data was greater in meningiomas classified as World Health Organization Grade II (atypical) than those classified as Grade I (benign). Immunoscreening using anti-ZIC antibodies revealed that 23 out of 23 meningioma cases were ZIC1/2/3/5-immunopositive. By comparison, nuclear staining by the anti-ZIC4 antibody was not observed in any meningioma case, but was strongly detected in all four medulloblastomas. ZIC-positive meningiomas included meningothelial, fibrous, transitional, and psammomatous histological subtypes. In normal meninges, ZIC-like immunoreactivities were detected in vimentin-expressing arachnoid cells both in human and mouse.

**Conclusions:**

ZIC1, ZIC2, and ZIC5 are novel molecular markers for meningiomas whereas *ZIC4 *expression is highly selective for medulloblastomas. The pattern of *ZIC *expression in both of these tumor types may reflect the properties of the tissues from which the tumors are derived.

## Background

Meningiomas are primary central nervous system tumors derived from arachnoidal (meningothelial) cells [reviewed in [1-3]]. Meningiomas are the most common type of benign intracranial brain tumor, with an annual incidence of approximately 2.3 to 6 per 100,000 persons [1]. They are classified into three World Health Organization (WHO) grades: benign (Grade I), atypical (Grade II), and malignant (Grade III) [1] based on the degree of anaplasia, number of mitoses, and presence of necrosis [1,3]. Grade I, II and III meningiomas account for approximately 80%, 5% to 20%, and 1% to 2% of all meningiomas, respectively [1]. Further classification is possible based on histopathological types; for example, Grade I tumors include meningiothelial, fibrous, transitional (mixed), psammomatous, angiomatous, and secretory meningiomas [1]. Clinicopathological examination of meningioma has benefited from the discovery of several molecular markers such as vimentin and epithelial membrane antigen (EMA) [1]. The presence of these molecules in meningiomas may partly reflect the cellular properties of arachnoidal cap cells from which meningiomas are thought to derive.

Recently, Inoue et al. [4] revealed that developing meningeal cells in mouse produce Zic family zinc finger proteins. Zic proteins are known to play critical roles in animal development [reviewed in [5]]. In humans, mutations in *ZIC *genes are associated with congenital anomalies such as holoprosencephaly (medial forebrain dysgenesis), heterotaxy (left-right axis disturbance), and Dandy-Walker malformation (cerebellar dysgenesis) [reviewed in [5,6]]. In meningeal cell development, mouse Zic proteins are present in the primitive meninx (meningeal cell precursors), and a deficiency of Zic2 or Zic1/Zic3 results in impaired proliferation and differentiation of meningeal precursors [4]. These findings led us to hypothesize that ZIC proteins are present in meningiomas.

Previous studies have reported immunoreactivities to ZIC proteins in medulloblastoma, another type of brain tumor. *ZIC1 *is predominantly expressed in medulloblastoma [7,8], and *ZIC2 *expression is down-regulated in medulloblastoma compared to its mRNA level in normal cerebellum [9]. In normal tissue, *ZIC1 *is preferentially expressed in cerebellar granule neurons and their progenitors [8]. The abundance of the ZIC1 protein in medulloblastoma is considered to reflect the properties of the cell of origin, cerebellar granule neuron. However, the expression of the other *ZIC *genes (*ZIC2*, *ZIC3*, *ZIC4*, and *ZIC5*) has not been investigated in medulloblastoma or other brain tumors. A comparison of the expression of *ZIC *family members is required. But current studies are limited by the structural similarities and the cross-reactivity of the antibodies [10].

These observations led us to investigate the expression profiles of *ZIC *genes in brain tumors, focusing on meningiomas. We first examined the levels of *ZIC1-5 *mRNAs in various brain tumors. Then ZIC-like immunoreactivity was examined in meningiomas and other brain tumors. Our results indicate that the expression of *ZIC1*, *ZIC2 *and *ZIC5 *is a conserved feature of meningioma.

## Methods

### Human materials and animals

All sampling procedures for human brain tumors were performed according to the Ethics Guidelines for Human Genome/Gene Analysis Research published by the Japanese Ministry of Education, Culture, Sports, Science and Technology http://www.lifescience.mext.go.jp/files/pdf/40_213.pdf. C57BL/6J mice were purchased from Nihon SLC (Shizuoka, Japan). Animal experiments were approved by the Animal Experiment Committee of the RIKEN Brain Science Institute.

### RNA isolation and RT-PCR analysis

RNA samples were prepared from human brain tumors using TRIZOL (Invitrogen, Carlsbad, CA, USA). Total RNA from human whole brain, liver, lung, kidney, and kidney tumor was purchased from Clontech (Mountain View, CA, USA). All RNA samples were treated with RNase-free DNase I (Promega, Madison, WI, USA) before the reverse transcription reaction. Reverse transcription and PCR were performed using ThermoScript reverse transcriptase (Invitrogen) and TaKaRa Ex Taq Hot Start Version (TAKARA BIO, Shiga Japan), respectively. The accession numbers, primer sequences, the size of PCR products, and number of PCR cycles were as follows: *ZIC1 *(NM_003412), 5'-GGCCCGGAGCAGAGTAAT-3' and 5'-AGCCCTCAAACTCGCACTT-3' (229 bp, 26 cycles); *ZIC2 *(NM_007129), 5'-CCCTTCAAGGCCAAATACAA-3' and 5'-TGCATGTGCTTCTTCCTGTC-3' (218 bp, 26 cycles); *ZIC3 *(NM_003413), 5'-GCAAGTCTTTCAAGGCGAAG-3' and 5'-CATGCATGTGCTTCTTACGG-3' (225 bp, 28 cycles); *ZIC4 *(NM_032153), 5'-GCCCTTCAAAGCCAAATACA-3' and 5'-GCCCTCGAACTCGCATC-3' (172 bp, 28 cycles); *ZIC5 *(NM_033132), 5'-TCTGCTTCTGGGAGGACTGT-3' and 5'-GGGAATGTTTCTTCCGATCA-3' (252 bp, 28 cycles); and *ACTB *(NM_001101), 5'-CAACCGCGAGAAGATGACC-3' and 5'-TCCAGGGCGACATAGCACA-3' (324 bp, 22 cycles). Each PCR cycle consisted of 1 min at 94°C, 30 s at 62.5°C, and 1 min at 72°C. RT-PCR product was harvested during the log-linear phase of the amplification curve at the PCR cycles indicated above. The PCR products were separated by electrophoresis in a 2.0% agarose gel and quantified by densitometry of ethidium bromide-stained bands using ImageJ version 1.33 http://rsb.info.nih.gov/ij/. The transcript amounts were normalized to *ACTB*. The results are indicated as relative values to the transcript amount in whole brain total RNA. The means of the three independent RT-PCR experiments are indicated. The absence of amplified products in RNA samples that had not been reverse transcribed was confirmed at the cycles indicated (data not shown).

### Analysis of public microarray data

The meningioma microarray results of Keller et al. [11] were obtained from the Gene Expression Omnibus (GEO) repository ftp://ftp.ncbi.nih.gov/pub/geo/DATA/supplementary/series/GSE12530/GSE12530_RAW.tar; the data included 24 meningioma and two dura mater controls profiled on GE Healthcare/Amersham Biosciences CodeLink Human Whole Genome Bioarrays. Following probes (ID_REF) were subjected for the analysis: *ZIC1*, 228079; ZIC2, 56068; *ZIC3*, 387012; *ZIC4*, 456009; *ZIC5*, 80028. For each gene, expression level in meningioma was indicated relative to the expression level in normal dura mater.

### Protein immunoblots and immunohistochemistry

Human *ZIC1-5 *expression plasmid vectors were constructed in pcDNA3.1 vector (Invitrogen) that had been modified to contain three tandem hemagglutinin (HA) epitope tag-encoding sequences. The open reading frame sequences were obtained by PCR amplification of a human brain cDNA (Clontech) and a BAC clone (RP11-1148D4, BACPAC Resources Center, CHORI, Oakland, CA, USA) and subsequent reconstruction of the sequence-verified fragments.

The expression vectors were transfected into NIH3T3 cells or 293T cells using TransIT-LT1 transfection reagent (Mirus, Madison, WI, USA). For protein immunoblot, the transfected cells were lysed in sodium dodecyl sulfate - polyacrylamide gel electrophoresis (SDS-PAGE) loading buffer. The proteins were separated by SDS-PAGE and transferred to polyvinylidene di?uoride membranes. The membranes were first incubated with 5% skim milk in phosphate-buffered saline (PBS) containing 0.1% Tween20 (PBST) for 1 h to block non-specific binding, and then incubated with either rat monoclonal anti-HA tag antibody (3F10, 1:4000), rabbit polyclonal anti-Zic2 antibody [CXY2, [12]], mouse monoclonal anti-Zic1 antibody [ZC26, [8]], or rabbit polyclonal anti-ZIC4 antibody (ProteinTech Group, Chicago, IL, USA) diluted in the blocking buffer, at 4°C overnight. Membranes were then washed in PBST for 1 h, and incubated for 1 h with horseradish peroxidase-conjugated anti-rabbit, anti-rat, or anti-mouse antibodies (Jackson ImmunoResearch Laboratories, West Grove, PA, USA). After re-washing with PBST, the bound antibodies were detected using enhanced chemiluminescence Western blot detection reagent (GE Healthcare, Buckinghamshire, UK).

Immunofluorescence staining was performed as described previously [4,13]. Immunohistochemical staining of human brain tumor specimen was performed as described [8] and briefly described below. Formaldehyde-fixed, paraffin embedded sections (3 to 6 μm) were prepared using a standard procedure [8]. The antigen retrieval was performed by autoclaving the dewaxed, hydrated tissue specimens in 10 mM sodium citrate (pH 6.5) at 121°C for 5 min. The sections were further incubated in 0.3% hydrogen peroxide for 10 min to inactivate the endogenous peroxidase-like activity. Blocking of non-specific binding was performed by immersing the specimens in a PBST containing 1% skim milk and 2% normal goat serum for 30 min at room temperature. The primary antibodies used in the immunohistochemical staining were anti-ZIC (CXY2, ZC26, and anti-ZIC4) anti-vimentin (Nichirei Biosciences, Tokyo, Japan), anti-EMA (Dako, Glostrup, Denmark), and anti-chondroitin sulfate proteoglycan (CS56, Sigma-Aldrich, St. Louis, MO, USA).

The sections were incubated at 4°C overnight in blocking buffer containing appropriately diluted primary antibodies (CXY2, 1:2000; ZC26, 1:500; anti-ZIC4, 1:400; anti-vimentin, 1:300; anti-EMA, 1:4). The bound primary antibodies were detected by immunoperoxidase reaction using VECTASTAIN Elite ABC kit (Vector Laboratories, Burlingame, CA, USA) and 3,3'-Diaminobenzidine (DAB) as a substrate. The double labeling was carried out by sequential primary antibody incubation and detection using two different chromogens, DAB and DAB-Nickel. Digital images were obtained using the NanoZoomer Digital Pathology C9600 (Hamamatsu Photonics, Shizuoka, Japan) image scanner and analyzed using the NDPViewer software (Hamamatsu Photonics).

## Results

### Expression of *ZIC1-5 *in various types of brain tumors

**Figure 1:**
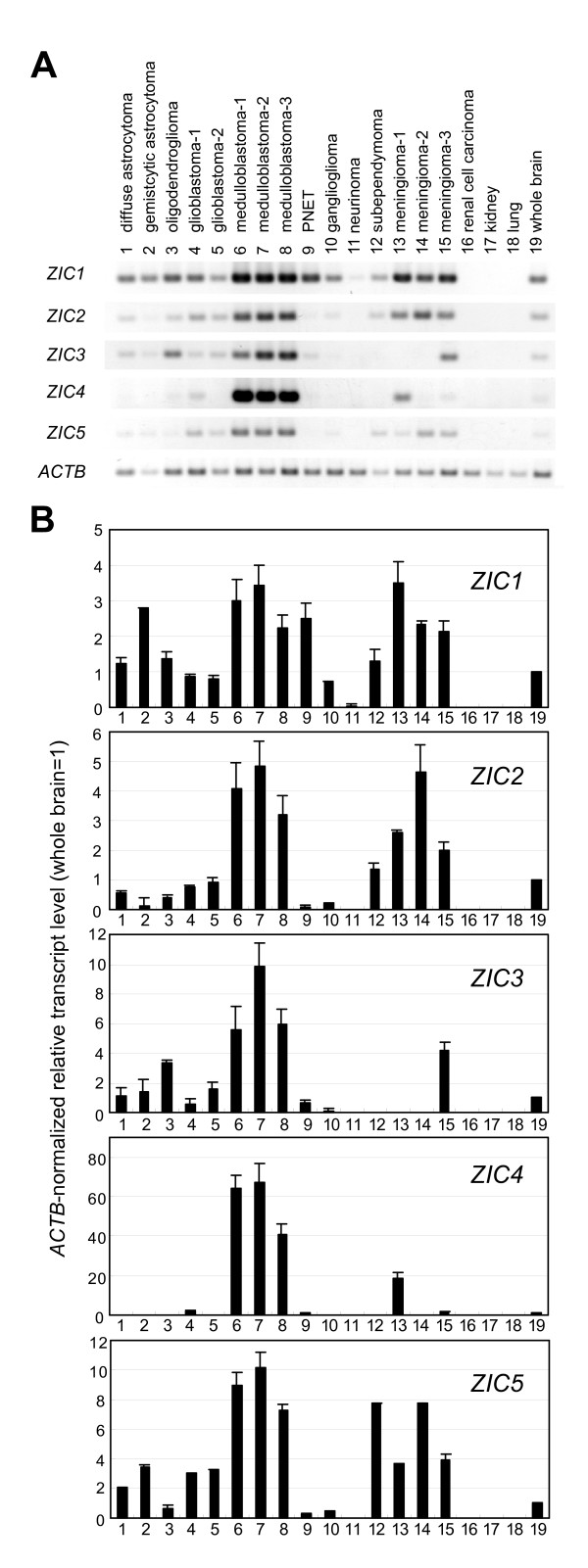
**RT-PCR analysis of *ZIC1--5 *expression in normal tissue and brain tumor tissue**. (A) Representative photographs of agarose gels showing RT-PCR products. (B) Relative amounts of *ZIC *transcripts. The transcript amounts have been normalized to *β *actin (*ACTB*) so that the value for the whole brain equals 1.0. Each bar indicates the mean of three independent RT-PCR analyses. Error bars indicate the standard error of the mean. Note that the graphs in (B) indicate the relative transcript amount to those of brain. The relative abundance among the five *ZIC *transcripts may be partly represented by the difference in the band intensity in (A) together with the PCR cycle and product size differences (See *Methods*).

We first examined the mRNA levels of the five *ZIC *genes in various types of brain tumors including astrocytoma, oligodendroglioma, glioblastoma, medulloblastoma, primitive neuroectodermal tumor, ganglioglioma, neurinoma, subependymoma, and meningioma (Figure 1, Table 1). A semi-quantitative RT-PCR analysis was performed using *ZIC *gene specific primer sets and cDNA synthesized from brain tumor RNA. The most consistent feature of *ZIC *expression was the high level of all five *ZIC *transcripts in the three medulloblastoma cases, when compared to their level in whole brain; the relative amounts varied from 2- to 3-fold for *ZIC1 *to 40- to 70-fold for *ZIC4*. In the three meningioma cases, the *ZIC1*, *ZIC2 *and *ZIC5 *mRNA levels were 2- to 3.5- fold, 2- to 4.7- fold, and 3- to 8-fold higher, respectively, than those in the whole brain. The *ZIC3 *and *ZIC4 *mRNA levels were higher in meningioma than in whole brain in one out of the three cases.

**Table 1:** Clinicopathological information on the tumors examined in the RT-PCR analysis

**No**.	Age	Gender	Location	Histological type
1	28	F	Right frontal lobe	Diffuse astrocytoma
2	43	F	Right frontal lobe	Gemistocytic astrocytoma
3	50	M	Left frontal lobe	Oligodendroglioma
4	67	F	Bilateral frontal and parietal lobes	Glioblastoma
5	57	M	Left frontal lobe	Glioblastoma
6	3	M	Cerebellar vermis	Medulloblastoma
7	12	M	Cerebellar vermis	Medulloblastoma
8	19	F	Cerebellar hemisphere	Medulloblastoma
9	30	M	Right parietal lobe	Supratentorial PNET
10	17	F	Right temporal lobe	Ganglioglioma
11	50	F	Left cerebellopontine angle	Vestibular neurinoma
12	70	M	Left frontal lobe (intraventricular)	Subependymoma
13	77	M	'Convexity'	Meningotheliomatous meningioma
14	57	M	'Falx'	Fibrous meningioma
15	66	F	'Parasagittal'	Meningioma
16	58	F	Right kidney	Fuhrman grade II Renal cell carcinoma

### Expression of *ZIC1-5 *in meningioma with different histological grades

**Figure 2:**
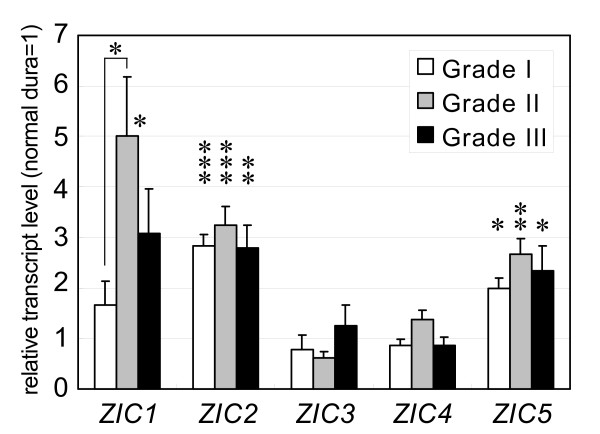
**The relative mRNA level of *ZIC1--5 *in meningioma groups of different histological grades**. The transcript levels were deduced from a recent microarray analysis [11] that includes eight each of Grade I (benign, open bar), Grade II (atypical, gray bar), and Grade III (anaplastic, malignant, black bar) meningiomas. The transcript levels in meningioma are indicated as relative to the level in normal dura mater. Each bar indicates the mean of eight independent tumor specimens. Error bars indicate the standard error of the mean. The asterisks above bars indicate the statistical significance of the difference between the meningioma classes and the dura maters, and the asterisk above the bracket indicates that of the *ZIC1 *expression difference between Grade I and Grade II meningiomas. *, *P *< 0.05; **, *P *< 0.01; ***, *P *< 0.001 in Welch's t-test.

We next evaluated *ZIC1-5 *mRNA levels in meningioma of various histological grades. For this purpose, we utilized a recent study in which a total of 24 meningioma cases and two normal dura mater tissue samples were subjected to microarray analysis [11]; the cases comprised eight benign (WHO Grade I), eight atypical (WHO Grade II) and eight malignant (WHO Grade III) tumors. For each *ZIC *gene, the mRNA level in meningioma relative to the level in normal dura mater was deduced from the published data (Figure 2). The mRNA levels of *ZIC1*, *ZIC2 *and *ZIC5 *were higher in meningioma than in dura mater. *ZIC1 *levels were significantly higher in Grade II tumors (5.0 fold) than in Grade I tumors (1.7 fold) (*P *= 0.027, Welch's t-test).

### Immunoscreening of ZIC proteins in brain tumor specimens

**Figure 3:**
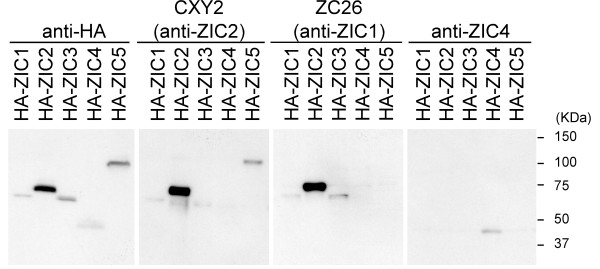
**Specificity of the anti-ZIC antibodies**. Protein immunoblot analysis. N-terminally HA epitope-tagged ZIC proteins were produced in 293T cells and were detected by CXY2, ZC26, anti-ZIC4 and anti HA-tag antibodies.

For the immunohistochemical detection of human ZIC proteins, we used three anti-ZIC antibodies. Anti-Zic2 polyclonal antibody (CXY2) and anti-Zic1 monoclonal antibody (ZC26) recognize the C-terminal regions of mouse Zic2 and Zic1, respectively. Anti-ZIC4 polyclonal antibody was raised against a peptide corresponding to human ZIC4 sequence. We first checked each antibody's specificity by protein immunoblot analysis (Figure 3) and immunofluorescence staining (Additional file 1) of the N-terminally epitope-tagged human ZIC1-5 proteins produced in mammalian cells. In both assays, CXY2, ZC26, and anti-ZIC4 recognized ZIC1/ZIC2/ZIC3/ZIC5, ZIC1/ZIC2/ZIC3, and ZIC4, respectively. The combinatorial use of these three antibodies was expected to cover all five ZIC proteins.

We then applied these antibodies to the immunostaining of paraffin-embedded brain tumor sections from patients, in order to examine whether ZIC-like immunoreactivity is present in meningioma (Table 2). Both CXY2 and ZC26 gave ZIC-like signals in the cell nuclei of all 23 meningioma cases. The staining intensity was generally higher for CXY2; but there was no obvious difference between CXY2 and ZC26 in terms of the distribution of the signals across tissues (data not shown). By contrast, anti-ZIC4 staining did not produce clear nuclear staining in the meningioma cases. The anti-ZIC4 antibody generated weak cytoplasmic staining in a subset of cells (data not shown). We excluded the cytoplasmic staining from the current analysis because we did not observe any obvious cytoplasmic localization of epitope-tagged ZIC4 protein in cultured cells (Additional file 1). All three anti-ZIC antibodies gave clear nuclear signals in the four medulloblastoma cases (Table 2), consistent with the RT-PCR results.

**Table 2:** ZIC-like immunoreactivities in brain tumors

	CXY2	ZC26	ZIC4 nuc_stain*
Grade I Meningioma	++ (12/22)	++ (2/22)	- (22/22)
	+ (7/22)	+ (14/22)	
	± (3/22)	± (6/22)	

Grade II Meningioma**	++ (1/1)	++ (1/1)	- (1/1)

Grade III Meningioma**	++ (1/1)	+ (1/1)	- (1/1)

Medulloblastoma	++ (4/4)	++ (4/4)	++ (4/4)

Glioblastoma		± (1/4)	
	- (4/4)	- (3/4)	- (4/4)

Hemangioblastoma	± (2/4)	± (1/4)	
	- (2/4)	- (3/4)	- (4/4)

Primitive neuroectodermal tumor	+ (1/1)	+ (1/1)	- (1/1)

Atypical teratoid rhabdoid tumor	+ (1/1)	+ (1/1)	- (1/1)

Intensity of the staining: -, none; ±, low; +, medium; ++, high. *, Although ZIC4 antibodies gave both nuclear and cytoplasmic staining, only the nuclear staining (nuc_stain) were evaluated. **, these two specimens derive from the same patient at different stages.

**Figure 4:**
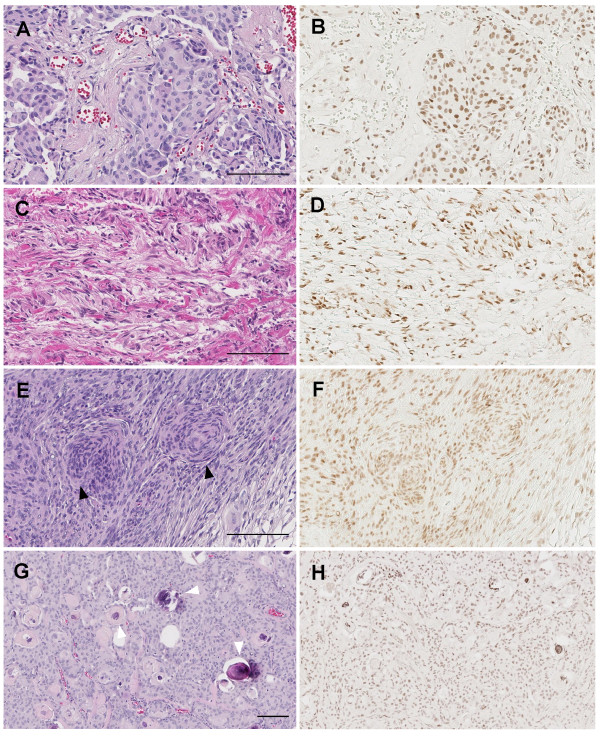
**Immunostaining of benign meningioma with CXY2 antibody**. The representative immunostaining images for each histological subtype. (A, B) Menigothelial meningioma. (C, D) Fibrous meningioma. (E, F) Transitional meningioma; characteristic whirling (black arrowheads) are observed. (G, H) Psammomatous meningioma; some psammoma bodies are indicated by white arrowheads. (A, C, E, G) Hematoxylin and eosin staining. (B, D, F, H) Immunostaining with CXY2 antibody. Scale bar, 100 μm.

**Figure 5:**
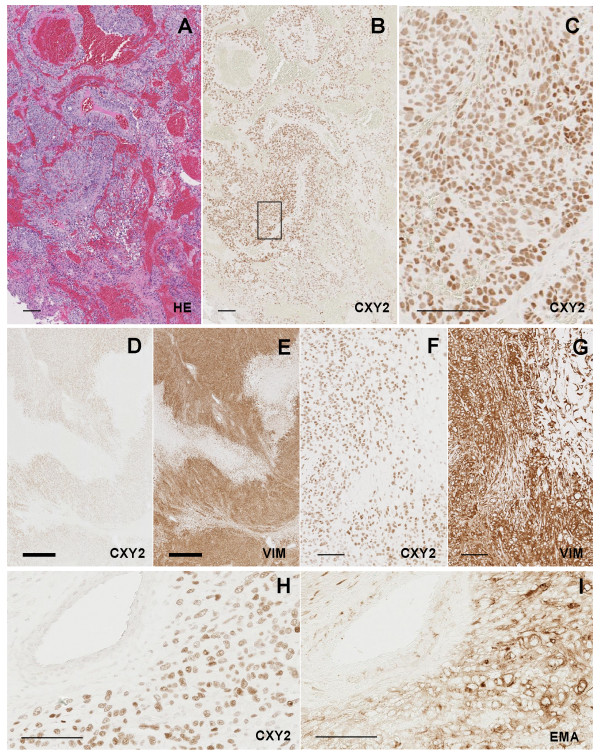
**Detection of ZIC-like immunoreactivities in atypical and malignant meningioma**. (A--C) Atypical meningioma with partially "blastic changes". (D--I) Anaplastic meningioma with geographic necrotic patterns. Each set of sections (A, B), (D, E), (F, G), and (H, I) is comprised of neighboring sections. (C) is the higher magnification of the boxed region in (B). (A) Hematoxylin and eosin staining. (B, C, D, F, H, J) Immunostaining with CXY2 antibody. (E, G) Immunostaining with anti-vimentin (VIM) antibody. (I, K) Immunostaining with anti-EMA antibody. Thick scale bar, 1 mm; thin scale bar, 100 μm.

The histological subtypes of meningiomas included in this study were meningothelial meningioma (Figure 4A, B), fibrous meningioma (Figure 4C, D), transitional (mixed) meningioma (Figure 4E, F), psammomatous meningioma (Figure 4G, H), atypical meningioma (Figure 5A-C) and anaplastic meningioma (Figure 5D-I). CXY2/ZC26-positive signals were detected broadly in the tumor cell nuclei of each histological type (CXY2: Figure 4B, D, F, H, and Figure 5B, C; ZC26: data not shown), and overlapped with two meningioma molecular markers, vimentin and EMA (Figure 5D-I). There was no discernable difference between the intensity of staining among the different histological grades.

We performed immunohistochemical staining of other types of brain and intracranial tumors as references (Table 2). In glioblastoma multiforme, no ZIC-like signals were detected, with the exception of one of the four cases that showed a weak signal for ZC26. Two out of four hemangioblastomas stained weakly with CXY2. A primitive neuroectodermal tumor and an atypical teratoid rhabdoid tumor were CXY2-immunopositive.

### ZIC proteins can be detected in normal arachnoid cells

**Figure 6:**
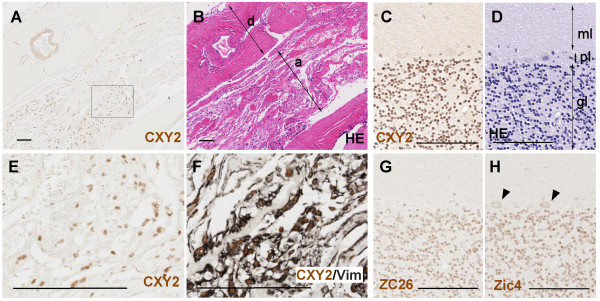
**Distribution of ZIC proteins in normal tissues**. Human adult-derived normal tissues were examined. (A, B, E, F) Meningeal membrane. (A, B, F) is comprised of neighboring sections. Image in (E) is a higher magnification of the boxed region in (A). Immunostaining with CXY2 (A, E), CXY2/vimentin (F), and hematoxylin and eosin staining (B) are shown. ZIC-like immunoreactivities are indicated as brown signals in (A, E, F). Vimentin-like signals are indicated by black in (F). Most Zic-like signals in cell nuclei are surrounded by the cytoplasmic Vimentin-like signals. a, arachnoid layer; d, dura mater. Scale bar, 100 μm. (C, D, G, H) Cerebellum. Immunostaining with CXY2 (C), ZC26 (G), and anti-ZIC4 (H), and hematoxylin and eosin staining (D) are shown. ZIC-like immunoreactivities are indicated as brown signals in (C, G, H). The majority of the cell nuclei in the granule cell layer are commonly stained with the three anti-Zic antibodies. In (H), the arrowheads indicate the cytoplasmic staining of the cells in Purkinje cell layer. The derivation of the cytoplasmic signals by the anti-ZIC4 antibody staining is unknown. gl, granule cell layer of cerebellum; ml, molecular layer of cerebellum; pl, Purkinje cell layer. Scale bar, 100 μm.

To examine whether the presence of ZIC proteins in meningioma relates to the tumor's proposed histogenesis, we examined the localization of ZIC-like signals in normal tissue adjacent to the tumor tissue. When we examined the meningeal tissues, CXY2-positive signals were detected in arachnoid cells that express vimentin, but were not detected in the dura mater (Figure 6A, B, E, F). ZIC4-positive signals were not detected in arachnoid or dura mater cells (data not shown). By comparison, all three of the anti-ZIC antibodies gave clear signals in the cell nuclei of cerebellar granule neurons (Figure 6C, D, G, H), from which medulloblastomas are considered to be derived.

**Figure 7:**
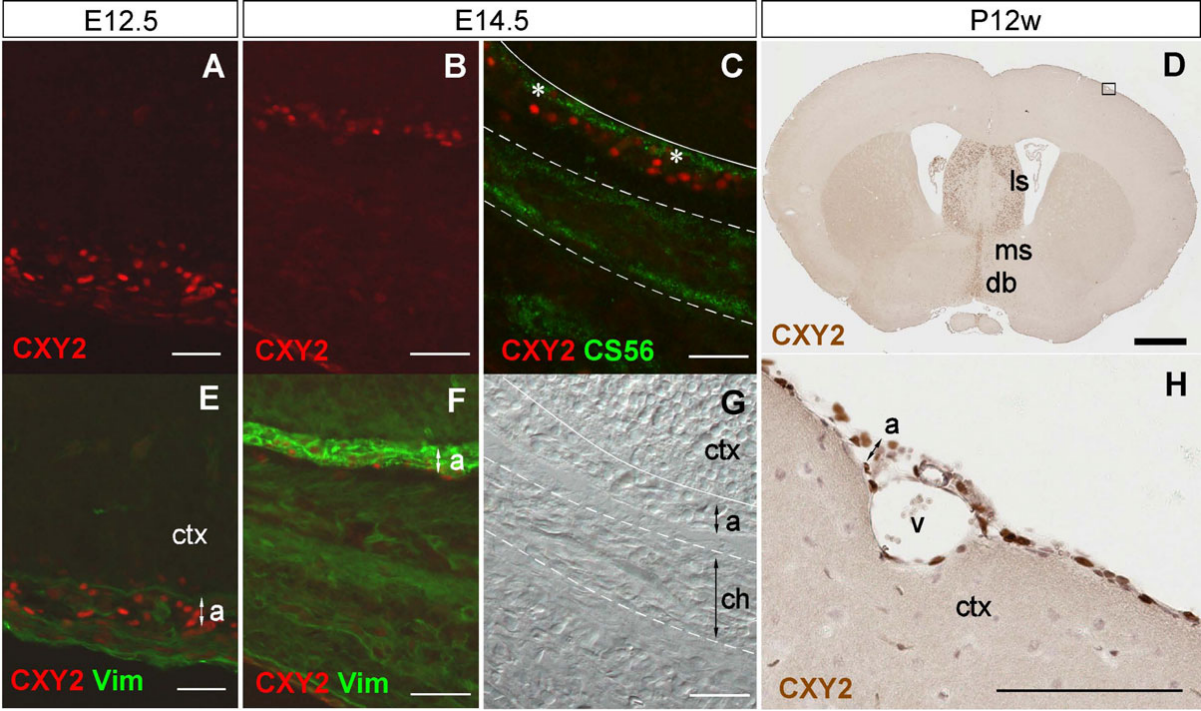
**Distribution of Zic proteins in mouse meningeal membrane**. Mouse sections were derived from coronal sections through the forebrain at embryonic day 12.5, E12.5 (A, E), embryonic day 14.5, E14.5 (B, C, F, G) or 12 weeks after birth, P12w (D, H). Images (A, E), (B, F) and (C, G) indicate the same regions respectively. Image in (H) is a higher magnification of the boxed region in (D). Immunostaining with CXY2 (A, B, D, H), CXY2/vimentin (E, F), and CXY2/CS56 (C), and differential interference contrast image (G) are shown. Zic signals are indicated by red in (A--C, E, F), and brown in (D, H). The colour green indicates CS56 (C) and vimentin (E, F) immunoreactivities. In (C), the ZIC-like and CS56 immunoreactivities overlapped within the arachnoid layer that expresses vimentin; whereas, only CS56--positive signals were detected within the chondrogenic area, between the two broken lines. The white line indicates the interface between meningeal tissue and cerebral cortex. CS56--positive signals were detected in the region that forms the basal lamina (asterisks). In the adult mouse brain, Zic proteins were still produced in the meningeal cells, as well as the lateral septal nucleus, medial septal nucleus, and diagonal band (D). The positive signals were detected in the arachnoid and perivascular cells (H). a, arachnoid layer; ch, chondrogenic region (prospective temporal bone); ctx, cerebral cortex; db, diagonal band; ls, lateral septal nucleus; ms, medial septal nucleus; v, vessel. Thick scale bar, 1 mm; thin scale bar, 100 μm.

We also explored the distribution of Zic proteins in meningeal cells during development, using mouse as a model. Mouse Zic proteins are present in meningeal cell progenitors as early as when the primitive meninx tissue appears (Figure 7A, [4]). CXY2-positive cells in the meningeal layer were vimentin-positive along the course of development (Figure 7E, F). After chondrogenic differentiation of the outer layers had occurred (Figure 7B, C, F, G), CXY2-positive signals were interposed between the neuroepithelial cells and the chondrogenic region, as delineated by CS56 staining (indicating the distribution of chondroitin sulfate proteoglycan, Figure 7C, G), and were limited to the arachnoid layer. The CXY2-positive signals were also detected in the mature arachnoid cells (Figure 7D, H), similar to our observation in the human adult meningeal membrane (Figure 6A, B, E, F).

## Discussion

### ZIC expression in meningioma and normal meningeal cells

The distribution of ZIC-like immunopositive signals in meningiomas may reflect the property of the arachnoid cells from which meningiomas arise. Since Zic proteins play an essential role in the proliferation of meningeal cell progenitors [4], we consider it possible that ZIC proteins are involved in the proliferation of meningioma cells. This is consistent with our observation that *ZIC1, ZIC2 *and *ZIC5 *mRNA levels are higher in meningiomas than in normal brain tissues. We suggest that studies to elucidate the involvement of ZIC genes in the meningioma tumor cell proliferation would be beneficial.

Antibodies against EMA, vimentin and Ki-67 have been used to provide histopathological differential diagnoses of meningioma and to estimate its malignancy grade [1]; however, no molecular marker that is specific for meningiomas has been described to date. Although additional studies in more cases of various brain tumors are needed, detection of ZIC proteins in meningioma will be helpful for diagnoses in extraaxial brain tumors.

In terms of meningeal expression of *ZIC *genes, a previous *in situ *hybridization study [4] indicates that mouse *Zic1 *and *Zic2 *are strongly expressed in the embryonic meningeal cells and its precursors. Our study confirms these results and clarifies that the expression of *Zic *genes continues during development in the arachnoid cell lineage in mouse. We detected immunoreactive Zic/ZIC proteins in meningeal cells in both mouse and human adult brains. Although the physiological role of ZIC proteins in the adult meningeal cells has not been clarified, ZIC proteins might be involved in the maintenance of cell properties of differentiated arachnoid cells, analogous to their role in meningeal cell differentiation in mouse development.

### Differential expression of human ZIC genes in tumors

A major finding of this study is the differential expression of members of the human *ZIC *gene family in various brain tumor types. RT-PCR analysis revealed that *ZIC4 *expression is highly enhanced in medulloblastoma, in a sharp contrast to the expression levels in whole brain, while *ZIC1*, *ZIC2 *and *ZIC5 *are expressed both in the meningioma and medulloblastoma. The results of the immunoscreening are consistent with the RT-PCR results in that ZIC4-like nuclear staining was almost limited to the medulloblastoma cases. Although we detected low amounts of *ZIC4 *transcript in one of the three-meningioma cases, the immunoreactive ZIC4 protein level in this sample was below the limit of detection (data not shown). The distinctive expression profile of *ZIC4 *highlights its usefulness as a molecular marker for brain tumor pathology.

Interestingly, in a cohort of patients with small cell lung carcinoma, one of the most aggressive tumors known, autoantibodies to ZIC2 are present in 28% of patients, and the presence of these autoantibodies is associated with less aggressive clinical parameters such as a better response to initial therapy [14]. Autoantibodies in small lung cell carcinoma patients show cross-reactivity among ZIC1, ZIC2, and ZIC4 proteins [15], suggesting that immunoreactivities of the autoantibodies are directed primarily against the conserved zinc finger domains of ZIC [16]. Furthermore, ZIC autoantibodies have been detected in patients with both small cell lung carcinoma and paraneoplastic neurological syndrome, although it is not clear whether the immune response to ZIC proteins is pathogenically related to the development of the neurological syndrome [16]. In small cell lung carcinoma, it is important that the expression of ZIC proteins, and the usability of ZIC autoantibodies as a diagnostic tool, are further clarified. Therefore, comprehensive studies that examine each of the five members of the ZIC family proteins, and their corresponding autoantibodies are required.

Our findings, together with those of previous studies [8-10], demonstrate the importance of ZIC proteins in clinical oncology. Future studies will require improved ZIC protein- or anti-ZIC antibody-detection systems. We suggest that the anti-ZIC antibodies and the full-length ZIC1-5 expression vectors described in this study will be useful for this purpose.

## Conclusions

The main results can be summarized as follows:

*1) ZIC1*, *ZIC2*, and *ZIC5 *mRNA levels are high in meningiomas.

2) All *ZIC *genes are abundantly expressed in medulloblastomas.

*3) ZIC1 *mRNA levels are higher in Grade II than in Grade I meningiomas.

4) ZIC1/2/3/5-like immunopositive signals are detected in most meningiomas irrespective of their histological type.

5) ZIC1/2/3/5-like immunopositive signals are detected in the arachnoid cell lineage.

6) ZIC1/2/3/5 and ZIC4-like immunopositive signals are detected in both human cerebellar granule neurons and medulloblastomas.

## Abbreviations

ACTB: β actin; DAB: 3,3'-Diaminobenzidine; EMA: epithelial membrane antigen; HA: hemaglutinin; Zic: Zinc finger protein of the cerebellum.

## Competing interests

The authors declare that they have no competing interests.

## Authors' contributions

JA conceived the idea of the study, performed the RT-PCR and protein immunoblot experiments, analyzed the data, and drafted the manuscript. YN, YSO, and MH carried out immunostaining. NY participated in the design of the study, provided the materials, analyzed the data, and drafted the manuscript. All authors read and approved the final manuscript.

## Pre-publication history

The pre-publication history for this paper can be accessed here:

http://www.biomedcentral.com/1471-2407/10/79/prepub

## Supplementary Material

Additional file 1 **Immunofluorescence staining of the NIH3T3 cells producing HA-ZIC1-5 proteins. **The expressed proteins and the antibodies used for immunostaining are indicated at the side and top of the panels, respectively. All of the human ZIC proteins were detected in the cell nuclei. Click here for file
