# Supplementary figures and images for: Expression of ZIC family genes in meningiomas and other brain tumors
Source: BMC Cancer. 2010 Mar 3;10:79. doi: 10.1186/1471-2407-10-79 (PMC2838823; doi:10.1186/1471-2407-10-79)

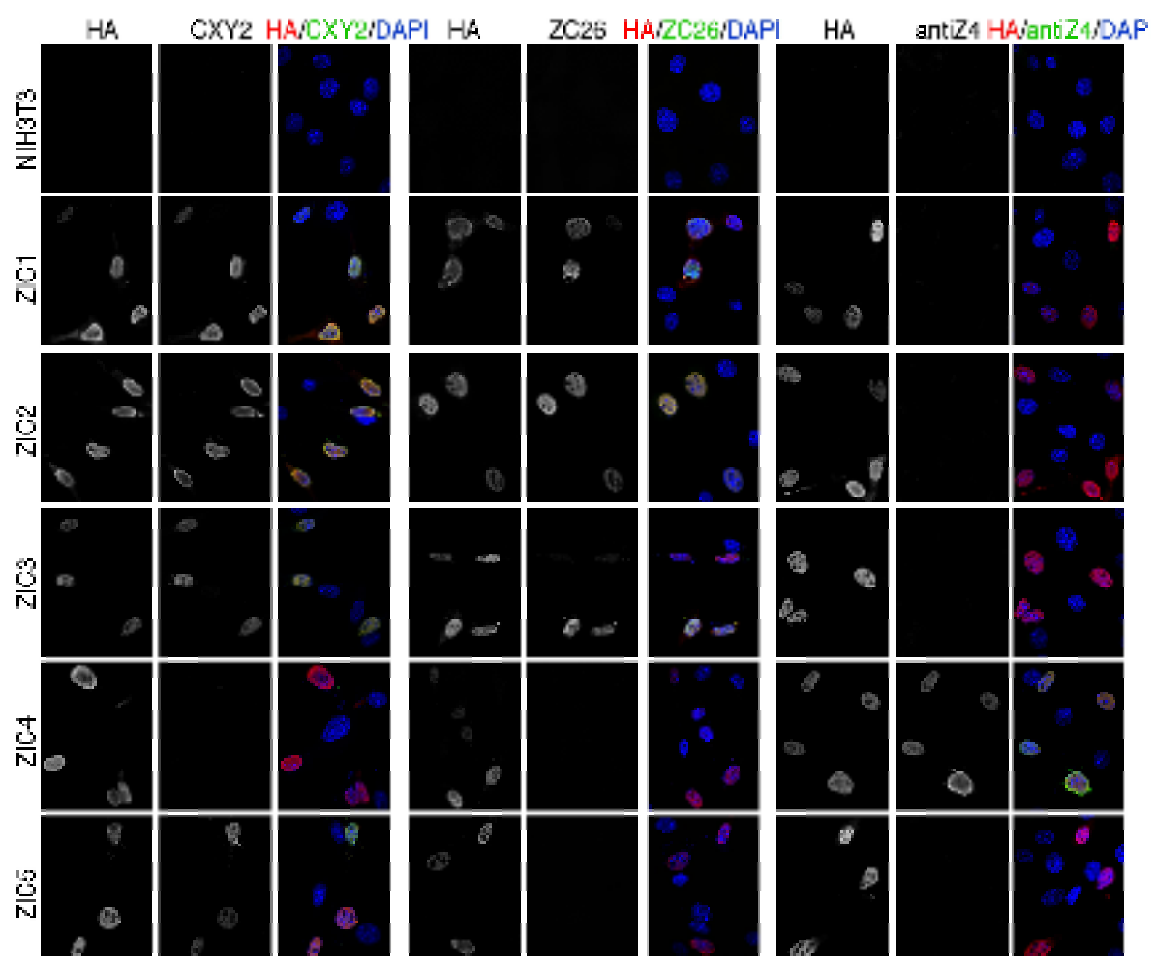

Supplemental Figure 1

Supplement: Additional file 1 — Immunofluorescence staining of the NIH3T3 cells producing HA-ZIC1-5 proteins. The expressed proteins and the antibodies used for immunostaining are indicated at the side and top of the panels, respectively. All of the human ZIC proteins were detected in the cell nuclei. [file 1471-2407-10-79-S1.PDF]
